# Early Risk Factor Prediction in Chronic Kidney Disease Diagnosis Using Feature Selection and Machine Learning Algorithms

**DOI:** 10.1055/a-2797-4380

**Published:** 2026-02-13

**Authors:** Chowdhury Nazia Enam Prima, Martti Juhola

**Affiliations:** 1 Data Science Research Center, Faculty of Information Technology and Communication Sciences, Tampere University, Tampere, Finland

**Keywords:** chronic kidney disease, early risk prediction, feature selection, machine learning algorithms

## Abstract

**Background:**

Chronic kidney disease, CKD in short, is a kind of long-term kidney illness in which rapid deterioration of kidney function is observed over a period of time. Unlike other organs, this damage in kidney function cannot be recovered and reversed as well. Moreover, in its early stages, asymptomatic renal disease is highly prevalent, making early identification with conventional clinical approaches difficult. Thus, early and accurate detection of risk factors is a very challenging step in CKD diagnosis.

**Objectives:**

This research work showed earlier and effective identification of risk factors using notable feature selection techniques for the enhancement of patient care. It also aimed at the improvement of predictive diagnosis of CKD employing different supervised and ensemble machine learning classifiers.

**Methods:**

A CKD-focused dataset consisting of 1,032 patient records and 14 features was used for this research purpose. This research emphasized on identifying the risk factors of CKD using feature importance (for tree-based model) with sequential feature selector and ReliefF algorithm as feature selection process. Based on the ranking for both feature selection techniques, the top 10 features were identified. Then utilizing those features, the classifiers such as random forest, support vector machine, Naïve Bayes, decision tree, logistic regression, gradient boosting, *K* -nearest neighbors, and ensemble classifier voting technique were trained using stratified 5-fold and grid-based search cross-validation techniques. After that, their performances were assessed using evaluation measures, i.e., accuracy, F1 score, precision, recall, training loss, test loss, bias, and AUC, to classify the individual having presence or absence of CKD.

**Results:**

The feature selection algorithms selected the significant data-driven top 10 features. Based on the ranking for both feature selection procedures, hemoglobin is determined to be the significant risk factor among these features. For both feature selection techniques, all the classifiers showed their best performance, having 86 to 98% of accuracy, AUC value of over 0.96 to 1.00, and bias value of 0.003 to 0.103. All the classifiers showed a very good trade-off between false positives and false negatives, with precision, recall, and F1 score ranging from 92 to 98%, 90 to 99%, and 93 to 98%, respectively, using feature importance with SFS. In both cases of the feature selection techniques, gradient boosting outperformed all other algorithms in terms of accuracy, precision, AUC, recall, F1 score, specificity, and bias.

**Conclusion:**

To conclude, in the suggested methodology the feature selection algorithms effectively identified the prominent features based on their importance, and the pipeline demonstrated a good performance in diagnosing individuals at risk of CKD development. Some of the classifiers showed their effectiveness in CKD prediction using the selected features by achieving higher accuracy, F1 score, precision, recall, AUC, specificity, and lower bias to ensure the diagnostic performance. Therefore, it can be inferred that this proposed methodology, combining the power of these eight machine learning models with two efficient feature selection approaches, demonstrated that people at risk of this nephrological condition can be detected earlier, more accurately identifying increased risk factors than with conventional methods. This holds a great promise toward enhancing healthcare judgment and eventually ensuring treatment for patients.

## Introduction

Chronic kidney disease, CKD in abbreviated form, is a long-lasting and degenerative nephrological disease in which kidney function deteriorates throughout a span of time. By 2040, it is anticipated that CKD will rank fifth globally in terms of years lost due to mortality. 1 This essential excretory organ eliminates metabolic byproducts and extra fluids from the body. Therefore, it is necessary to assess renal function for treating patients with renal disease or other conditions that impair renal function, evaluating the kidney's reaction to treatment, and monitoring the disease's progression. The most well-known factors influencing the progression of CKD involve rising prevalence of hypertension, cardiovascular disease, diabetes along with aging, and rising population particularly in countries with developed economies. 2 Since CKD affects up to one in three diabetics and one in five hypertensive individuals in countries with high incomes, it has been suggested that emphasizing diabetes and cardiovascular disease management could mitigate the rising burden of CKD. 3 As several factors mentioned above contribute to this progression, examinations alone may not be enough to prevent the disease from worsening. Determining creatinine (CR) content, estimated glomerular filtration rate (eGFR), albumin content in urine and blood, index of blood urea nitrogen (BUN), and ratio of 24-hour urine albumin–creatinine in the blood tests is frequently used to diagnose CKD. 4 However, medical treatments like dialysis or transplants are costly, notably in countries with limited resources. An estimated 850 million people worldwide have this nephrological condition where many of these people belonging to lower to middle economy lack access to kidney disease screening, prevention, or patient care. 5 Thus, considering the sheer number of healthcare data, predictive analytics in healthcare is highly essential for facilitating CKD prognosis and management.

Minimizing healthcare expenses, optimizing patient outcomes, and preventing the progression of this nephrological condition all depend on appropriate and early detection. Since asymptomatic kidney disease is highly prevalent in its early 1 to 3 stages where glomerular filtration rate (GFR) is greater than 30 mL/min/1.73 m ^2^ and also clinical disturbances like imbalance in electrolyte, metabolism, or fluids are not much experienced by patients, it is very challenging to make this crucial early identification utilizing only traditional clinical methods. 6 Instead of solely depending on conventional techniques, people having a risk of getting affected by this renal disease can be identified more efficiently by incorporating machine learning (ML) algorithms. Technological progression in deep learning (DL) and ML has created numerous opportunities for enhancing CKD's early detection. However, for accurate identification of risk factors, there can be several technical challenges such as handling noisy and multimodal data, dealing with confounding variables, and implementing unreliable models which can lead to inaccuracies and miscalculated values that might be sensitive in medical condition diagnosis. Driven by this motivation, our research work addressed this issue in case of clinical tabular data and demonstrated the effective identification of the top 10 risk indicators earlier using two noteworthy feature selection techniques, namely, feature importance (for tree-based model) with sequential feature selector and ReliefF algorithm.

We used a moderate sized (1,032 data records, 13 feature values, 1 target variable) multivariate clinical tabular dataset unlike the earlier works. Previously, majority of the authors used tiny CKD dataset (400 data records, 24 feature values, and 1 target variable) from UCI ML repository, which contains a substantial amount of missing values. This dataset is publicly available at https://archive.ics.uci.edu/dataset/336/chronic+kidney+disease . Using this dataset, a few authors employed various ML and DL approaches and feature selection methods to predict CKD detection. 7 8 9 Arif et al proposed a new methodology integrating Boruta algorithm as feature selection method with *K* -nearest neighbors ( *K* -NN) and Gaussian Naïve Bayes as ML methods for early detection of CKD using data on CKD from UCI. 7 Using CKD dataset from UCI ML repository, Majid et al explicitly focused on the usage of feature selector techniques like Info Gain Attribute Evaluator, Wrapper Subset Evaluator, and Ranker search platform combined with Best-First search mechanism and blending those with several ML and ensemble methods to significantly enhance the precision and accuracy of CKD prediction. 10 Nevertheless, in these papers the common objective was to improve diagnosis of CKD using feature selection techniques and ML methods. However, these papers did not specifically pinpoint and identify the crucial risk indicators by ranking them based on their importance. To cover these research gaps, the objectives of this research work include the following:

I. To explicitly investigate and rank top 10 data-driven important early risk factors using feature selection techniques like feature importance with sequential feature selector and ReliefF algorithm from the moderate-sized CKD-based dataset.
II. To utilize these risk factors for data-driven exploration of predictive diagnosis of CKD detection, implementing and systematically optimizing eight ML algorithms using stratified 5-fold and grid-based search cross-validation technique to explore the effect of hyperparameter tuning on model behavior.
III. To analyze the performance of ML classifier-based model and to find the best model using beyond regular evaluation metrics like AUC, accuracy, precision, recall, and F1 score, and also to report training loss, test loss, specificity, and bias unlike above-mentioned papers for emphasizing a diagnostic insight of model development.

Nevertheless, this proposed methodology comprehended the significant characteristics and features that influenced the predictions, and led to a reliable, scalable model that can be applied in clinical settings. The outcomes of this research may provide healthcare professionals with beneficial information, opening the door to more initiative-taking and individualized strategies for CKD management.

## Materials and Methods

**Fig. 1:**
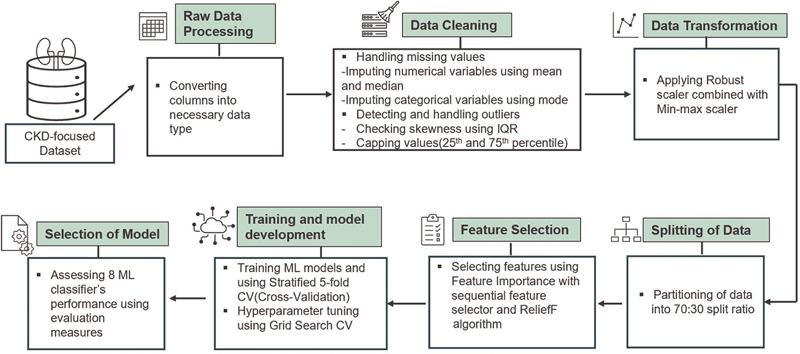
Schematic diagram of proposed methodology.

This research work followed a systematic workflow for identifying risk indicators earlier using feature importance with sequential feature selector, and ReliefF feature selection algorithm. This methodological choice directly addressed the gaps in previous research works, which typically relied on only using feature selection methods for improving the predictive performance of CKD diagnosis instead of explicitly identifying and ranking data-driven risk factors earlier. Here, an ablation experiment was also conducted separately using only the raw features to show the necessity of feature importance. A CKD-focused dataset used here for this research purpose was retrieved from one of the authors of reference. 10 11 Figure 1 is a diagrammatic illustration of different phases of suggested methodology.

After the proposed feature selection technique, our methodology utilized the identified top 10 risk factors for the training and development of classifiers for predicting CKD detection. The model was a collection of several supervised and ensemble learning algorithms. The classification efficacy of several methods was tested when ML algorithms like *K* -NN, decision tree (DT), random forest (RF), support vector machine (SVM), logistic regression, Naïve Bayes, gradient boosting (GB), and ensemble voting classifier (soft voting) were validated on the dataset. For ensuring better patient outcome, the effectiveness of the classifiers was evaluated using different evaluation measures such as AUC, accuracy, precision, recall, and F1 score, along with reporting training loss, test loss, specificity, and bias.

By substituting the desktop-based Python Jupyter Notebook with a cloud domain environment without requiring any setup, ML tasks were accomplished. All experiments were conducted on a virtual machine with two CPU cores (x86_64 architecture) and 12.67 GB RAM without any GPU acceleration in a 64-bit Linux (Google Colaboratory) environment running Python 3.12.11 with GCC 11.4.0. Pandas was used in this study for modeling and evaluation to facilitate data manipulation through a variety of functions, including the handling of missing data, data scaling, and more. Here, NumPy was utilized for dealing with arrays while Seaborn was used to explore and comprehend the data.

### Dataset

For this research study, a CKD-focused dataset containing 1,032 patient data was utilized for assessing the effectiveness of ML algorithms. The dataset was collected in Enam Medical College & Hospital in Bangladesh. The dataset was collected based on the questionnaires created by the authors of reference. 11 Here, each sample contained 13 predictive features showing the renal function tests required for CKD diagnosis. In addition, there was a categorical response variable named “Class” which was also the target variable. This binary variable had two separate values, 0 denoting patient records without CKD and 1 denoting records diagnosed with CKD. For better comprehension, summary of features (risk factors) included in the dataset is presented in Table 1 .

**Table 1:** Details of features (risk factors) in CKD dataset

Features	Denoted as	Interpretation
Blood pressure	Bp	Measured in mm/Hg
Specific gravity	Sg	Varies between 1,005 and 10,025 (higher values indicating higher risk)
Albumin	Al	Ranges from 0 to 5 (the higher, the better)
Sugar level	Su	Ranges from 0 to 4 (indicating 0 as low level and 4 as highest level of severity)
Red blood cells	Rbc	Denoted as 0 and 1 indicating abnormal and normal levels respectively
Blood urea	Bu	Measured in mg/dL
Serum creatinine	Sc	A high level is not a good indication
Sodium	Sod	Measured in unit mEq/L
Potassium	Pot	Measured in unit mEq/L
Hemoglobin	Hb	Less than 15 indicating kidney failure
White blood cells count	Wbcc	It includes numerical cells count
Red blood cells count	Rbcc	This value should not be less or higher than the normal
Hypertension	Htn	It indicates presence or absence of hypertension
Class	CKD or not	It denotes presence or absence of CKD

Abbreviation: CKD, chronic kidney disease.

### Dataset Preparation

This research work did not make use of the raw dataset. The unprocessed dataset was turned into a comprehensible structure by doing the necessary steps required for diagnosing CKD at early stages. The suggested methodology was employed on the processed dataset. For converting the raw data into understandable format, the data columns were transformed into necessary data types. Handling of missing values and outliers was also included in this data cleaning phase.

**Fig. 2:**
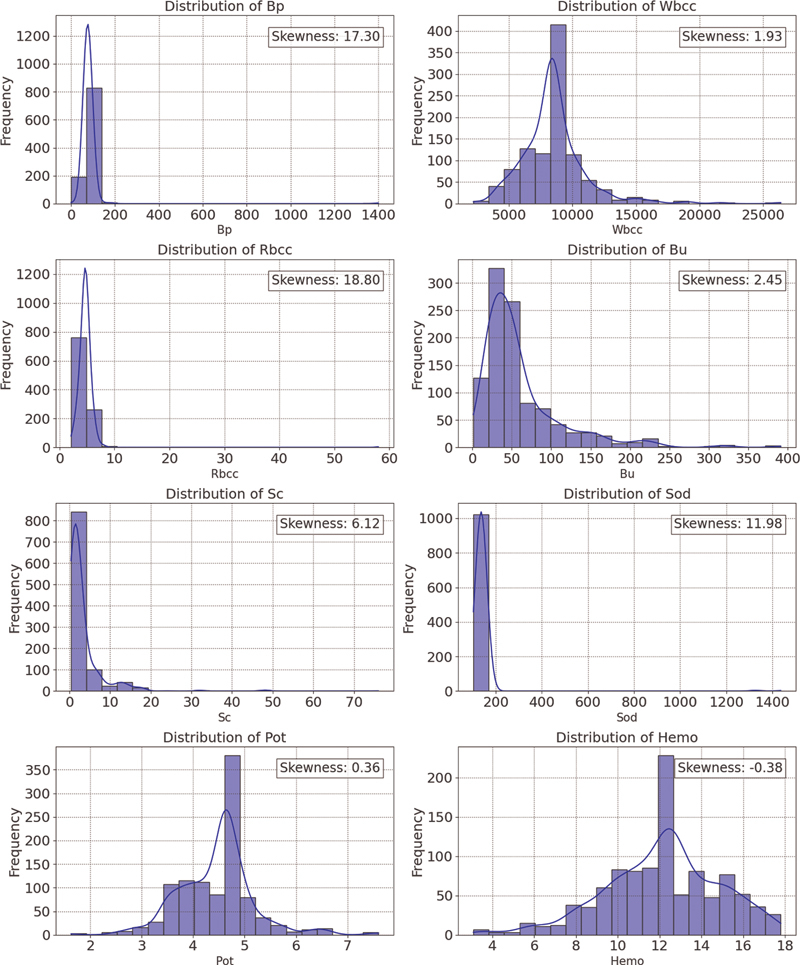
Distribution of the features in the dataset.

While handling missing values and outliers, the distribution and skewness of numerical columns were checked. Skewness is a measure of the degree of symmetry. A distribution is “positive-skewed” or “right-skewed” if the right tail is lengthier and more apparent and the distribution's mass is centered on the left. Imputation techniques like mean, median, and mode were used here depending on their skewness as shown in Fig. 2 , as these were suggested to work well with low proportion of missing values. 12 The strategies for imputing missing values in case of numerical features using mean or median, and imputing the missing values for categorical features using mode, were followed for handling missing values. Since “Sod” was the only column with low number, four missing values, and it also had a skewed distribution, the values were imputed using median. As interquartile range (IQR) was suggested to be less affected by skewness making it more robust for pinpointing outliers, therefore for detecting and handling outliers, **IQR** was used here. 13

Outliers were defined as data points which were greater or lower than the acceptable maximum and minimum values estimated. Following the detection of the outliers, the **capping** technique was used to cap the outliers with 5th percentile (lowest) and 95th percentile (highest) numbers by effectively confining extreme values within a reasonable range.

**Fig. 3:**
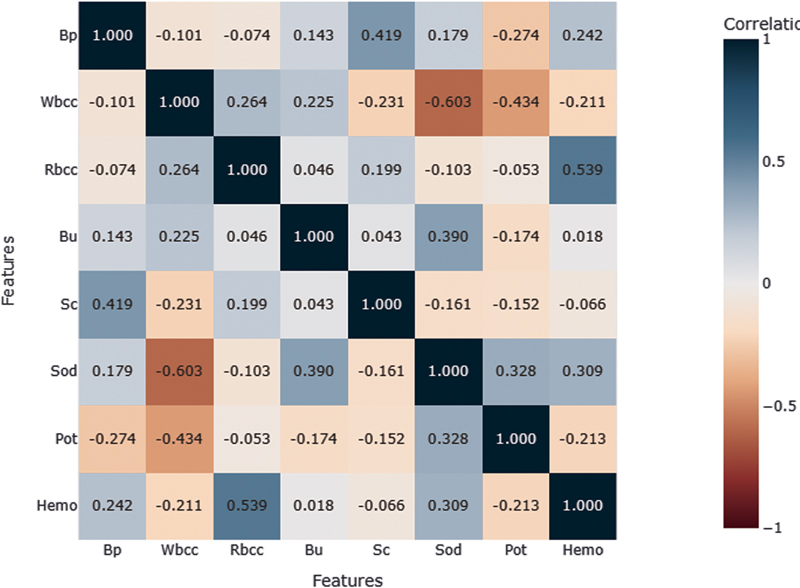
Correlation matrix of numerical features.

To provide useful insights for identifying risk factors and then develop the model, correlation matrix shown in Fig. 3 was generated. Here, significant positive associations were observed between Rbcc and Hemo, indicating good impact on each other with correlation coefficient 0.539. Bp and Sc had a positive correlation, having a correlation coefficient value of 0.419. Sod and Bu had a correlation coefficient value of 0.390 also indicating positive correlation between them.

#### Data Transformation

For scaling the features, **robust scaling and min-max scaling** were used together. Following the detection and capping of the outliers, this technique was employed because robust scaling can reduce the effects of outliers by scaling the features.

Because ML techniques like *K* -NN and support vector machine (SVM) are also sensitive to data scaling, min-max scaling was also crucial in this preprocessing step. Following robust scaling, features were scaled from 0 to 1 using min-max scaling.

#### Data Splitting

After the data transformation phase, following the random subsampling the dataset was divided into 70:30 ratio. It indicates that dataset's 70% was utilized for training and rest of 30% was allocated for the purpose of testing. To prevent information leakage, this splitting was completed prior to feature selection.

#### Feature Selection

Feature selection technique was performed on training set followed by the splitting of dataset. Then risk indicators were determined earlier using two feature selection approaches. The selected features were then applied to both test and training set. Feature selection methods which were used in this research work are: (1) feature importance with sequential feature selector and (2) ReliefF feature selection algorithm.

#### Feature Importance with Sequential Feature Selector

Feature importance allocated a score to each input feature in each model where it was used to determine feature's significance. A greater score suggested that the feature may influence the model, which was utilized for estimating a specific variable. To boost a predictive model's performance, a greedy technique often known as sequential feature selection (SFS) was used here, which repeatedly introduced or eliminated features from the dataset. Using a supervised feature selection method, this may either filter out irrelevant characteristics from dataset or pick significant features by incorporating them one after the other. 14

For improving performance of risk factors prediction of CKD diagnosis, feature importance with sequential feature selector was used to identify best top 10 features. Feature importance was computed for ML techniques such as RF, GB, and DT. It calculated the portion that each feature provided to the lowering of impurities in decision splitting. Subsequently, the mean significance was gathered and saved throughout these classifiers. SFS was used for approaches like logistic regression (LR), *K* -NN, SVM, and NB that did not explicitly offer feature importance. In this case, each picked feature was given similar importance labeling of 0 for not picked features and 1 for picked features. The features were then prioritized according to the average importance score, which was determined by adding up the feature importance from each classifier. Significant risk predictors were ranked and identified using this technique.

#### ReliefF Feature Selection Algorithm

The standard ReliefF approach was also used here as another feature selection technique. This is a revised version of the basic Relief method, which generally handles classifications of multiple classes with distorted, insufficient data. 15 The steps to find top 10 features using ReliefF algorithm were (1) initialization of feature weights, (2) random selection of instances, (3) finding nearest hits and nearest misses, (4) updating the feature weights, (5) repetition for randomly selected several instances, and (6) finalizing the weights for feature ranking. Here, initially a weight vector *W[X]* , containing all predictor weights, was initialized to 0. Then ReliefF chose *R _j_* instance randomly. After this, it started searching for nearest hits *H _k_* , *K* -NN that were in similar class, and searched for nearest misses *M _k_* (C), which were *K* neighbors from other *C* class. The features in the dataset were updated by this method according to their weights, which were dependent on how well they could differentiate between instances that were similar to one other but fell into separate classes denoted as misses or the same class denoted as hits.

Now the estimation for *W[X]* was updated for each feature depending on values of *R _j_* , nearest hits, and nearest misses. For hits, weight was decreased if *R _j_* and *H _k_* had same feature value. For misses, if a feature value was different for *R _j_* and *M _k_* (C), weight was decreased. Next, the average of each hit and miss was calculated. Each class's contribution to the misses was weighted according to the class's prior probability, or *P(C)* . It was required to be ensured that probability weights of misses' sum to one as in each step contribution for hits and misses needed to be [0,1]. For this, each predicted weight was divided with factor 1 – *P* (class ( *R _j_* )). It was then repeated for *n* times and *k* acted as user-defined parameters which controlled locality of the estimates. The steps were iterated multiple times and feature weights were updated. When the weight estimates were stabilized, final weights were used for ranking. This feature selection algorithm allowed to update the feature importance. More significant features were indicated by higher weights which could better distinguish instances between different classes and instances of same class. Finally, the top 10 relevant features were identified using this ReliefF approach and were trained on the dataset.

### Model Training and Hyperparameter Tuning

Following two notable feature selection techniques, eight different algorithms in the ML model were trained utilizing CV strategy and hyperparameters were tuned as well. This stage evaluated the model's strength for adapting to new data. To guarantee efficient performance of the classifiers on test set, the entire training set was further applied to retrain the finalized model incorporating those selected top features, after the identification of best performing model and hyperparameters. Here there is a brief overview of operating technique of eight algorithms.

#### Support Vector Machine

SVM generates output of optimal hyperplane in n-dimensional environment. 16 Finding the optimal decision boundary to maximize the margin and dividing the input data into many distinct classes are the key objectives of SVM. The separation among the most nearby datapoints of opposing classes is maximized by this optimum hyperplane. The probabilities of misclassifying the points are reduced by ensuring the maximization of distance. Margin is the space vertically measured between the hyperplane and the next data points. “Support vectors” are data points on the margins and help in determining the longest distance by going through the points. 17 In this study, linear and radial basis function (RBF) kernels were employed as hyperparameters. Additionally, hyperparameter C was utilized in the margin adjustment.

#### Decision Tree

This sequential and recursive model integrates different fundamental tests, each of which evaluates numeric attribute to a predetermined level. 18 To create a decision-tree model, it iteratively splits the data into subset based on most significant attribute at each phase following the splitting criteria. 19 Every subset goes through this process repeatedly, producing another leaf node until every instance in a node is of the same class, or a predetermined depth is achieved. The best attribute is chosen using the metric Gini impurity and entropy. This model utilized a greedy heuristic, which weighed possibilities at every training stage and chose the option that appeared best at that specific point. Three following hyperparameters were utilized for DT to regulate the tree's growth during training phase:

min_samples_split: It estimated the number of observations required at a certain node to be partitioned. A higher number of this parameter stops nodes from partitioning apart unless sufficient samples are present. This not only reduced overfitting but also limited the tree's complexity by forcing the tree to develop more prudently.
min_samples_leaf: This showed the minimum number of observations that, after a node split, must exist in the leaf node. The ultimate prediction was made by the end node.
max_depth: It determined the longest possible connection between the root and leaf nodes. It showed how many splits a tree could make before reaching a leaf node from the root node.

#### Random Forest

This robust approach aggregates many decision trees into an ensemble by utilizing bagging (easiest method for integrating classifiers) and randomness of features. RF utilizes one of the important concepts of bagging which is capacity to lower variances. This algorithm generates individual decision trees during training phase, which are linked with subsets of dataset. Each tree in the ensemble is constructed using feature randomness so that this can bring a unique perspective. During training, a random subset of features was chosen. The features were distributed randomly to each node; the tree was allowed to select from this subset, not from the entire dataset set. Using the highest voting results from each tree's target output, this model generates the single outcome which is a class target. The hyperparameters which were used for RF are given below:

n_estimators: This represented how many decision trees are present in the forest.
min_samples_split: It estimated the number of observations needed at a certain node to be partitioned.
min_samples_leaf: This showed the least number of observations that, after a node split, must exist in the leaf node.
max_features: This indicated the number of features which were taken into account at each splitting level.

#### Naïve Bayes

As the name implies, this probabilistic classifier is based on the Bayes' theorem, which operates under the belief that the occurrence of one feature does not indicate other features' occurrence. 20 Based on the likelihood that a particular data point belongs to each class, this classifier generated predictions and applied Bayes' theorem to determine the probabilities. There are different types of NB classifiers such as Gaussian, Bernoulli, multinomial NB etc. For this research purpose, Gaussian NB was used. According to Gaussian NB, every predictor can independently estimate the outcome variable, and the predictors follow Gaussian or normal distribution. During training phase, the classifier determined the prior likelihood of every class using the training data. The posterior probability of every class for a new sample was estimated subsequently during the prediction phase. It was further decided which class the data point belonged to depending on the obtained greater posterior probability.

#### *K* -Nearest Neighbors

*K-* NN is a supervised learning technique that estimates future outcomes by comparing the similarity of two input data points. It does not acquire knowledge right away from training set; hence, it also addressed as a lazy learner. By dividing input points into distinct classes, decision lines are formed using the distance metrics such as Euclidean, Manhattan, or Minkowski distance. It is required to identify the gap between other data points and input point to ascertain which are nearest to that input point. For this research work, hyperparameters named “n_neighbors” and “weights” were used. For “weights” parameter, both “uniform” and “distance” parameters were used to check the tuning of hyperparameters. These were additionally used to assess the performance of *K* -NN model.

#### Logistic Regression

LR is a well-liked supervised learning method used for predicting categorical outcomes. It derives estimations about the likelihood of events using independent variables. 21 The model is then trained for determining the best values for each independent variable to optimize the observed outcomes' probability. After training, the classifier uses the probability, i.e., *x̂ = σ(z)* , to establish a class label. The new input point is classified into a class based on a preset cutoff, which frequently takes 0.5 into account. When *x̂* is greater than or equal to 0.5, the instance is labeled as positive class 1; when *x̂* ≤ 0.5, the point is marked for negative class 0. After it has been trained, the model may predict the probability so that newly added input point will work in that specific class.

#### Gradient Boosting

This is an efficient ensemble ML technique which creates a single, highly accurate prediction model by repeatedly combining multiple poor models. Often, the process starts with a DT or by creating a base model. Since decision trees are so simple to interpret, they are frequently used with weak learners. The residuals (differences between actual variable and predicted variable) are determined by the technique that executes after the primary estimation. Every iteration fits the previous poor model's residuals to a new one, usually a decision tree. This model calculates this disparity among the target values and predicted variables utilizing a loss function. 22 To reduce the overall error, this obtained model is further trained to estimate the residuals. Subsequently, the predictions derived from the new model are integrated with those obtained from the old model. The following hyperparameters were employed in this research work for controlling boosting:

“learning_rate”: This parameter controlled each iteration's step size and specified the influence of every tree on the result. A reduced learning rate may yield to a more gradual yet delayed convergence. On the other hand, an increased number can lead to better performance because it is robust against over-fitting.
“n_estimators”: This variable denoted how many consecutive trees need to be modeled.
max_depth: It specified the longest possible connection between the root and leaf nodes and showed how many splits a tree can make before reaching a leaf node from the root node.

#### Voting Classifier

This ensemble classifier learns from collections of several models by estimating an outcome according to the models' highest possibility of identifying the preferred class. For voting purposes, two or more sub-models must be created where each of which generates estimations, for example, finding mode or mean of estimates. It enables a vote on the intended outcome for each sub-model. 23 There are two different types of this ensemble voting technique, i.e., hard voting and soft voting. In case of hard voting, the estimated output is the class having maximum number of votes. Soft voting is done by taking the mean of the probabilities of classes to make a final decision. For this research study, soft voting was used. Every model estimates a probability distribution across the potential classes for every test instance. A weighted mean of the probability distributions from each model is used to calculate the outcome. The weights may be determined by various factors or by evaluating each model's performance. The prediction is estimated for the class that has the greatest likelihood in the ultimate weighted mean. The optimal parameters selected for each classifier were considered while tuning hyperparameters. By aggregating predictions in an acceptable way, soft voting can improve the effectiveness of predictive ML approaches.

#### Cross-validation Techniques

The classifiers were trained using stratified 5-fold CV. The initial sample was divided into five equally sized smaller sets at random. After the model was trained on four of the folds, the other part of the dataset was kept for the purpose of testing. Additionally, Grid Search CV was used to specify a hyperparameter grid to be examined. However, stratified 5-fold was used for each combination of hyperparameters. The ideal collection of hyperparameters was chosen as the combination that yields the best average performance over all folds. 24 These methods assisted in fine-tuning models for finding optimal effectiveness and producing accurate performance predictions.

## Results

Classifiers' performances were assessed using evaluation measures such as accuracy, recall, precision, F1 score, ROC curve, and AUC. Additionally, training loss, test loss, specificity, and bias are calculated for each classifier for assessing their performance. Bias was calculated as the difference between the predicted positive rate and expected positive rate. Initially, the performance of the classifiers was assessed leveraging top 10 features using two notable feature selection techniques, and optimizing the hyperparameters implementing grid-based search CV. Then the performance of eight ML classifiers was presented using raw features without utilizing grid search CV to emphasize the importance of identifying important risk factors earlier and then utilizing those for model development. We also compared the performance of our proposed methodology with some previous studies.

### Performance Analysis Using Feature Importance and SFS

**Fig. 4:**
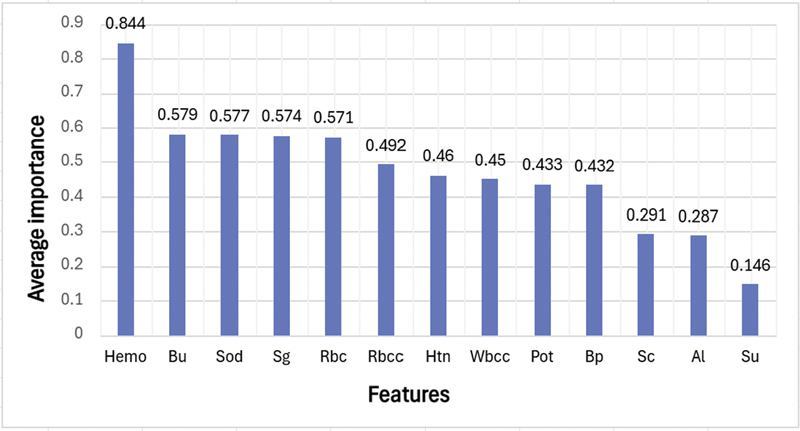
Average importance of all features using feature importance and sequential feature selection (SFS).

The top 10 features were selected by ranking the average importance in descending order using this feature selection technique. The average importance obtained for 13 features except the target class “CKD” is shown in Fig. 4 .

Based on the ranking, selected top 10 features were: “Hemo,” “Bu,” “Sod,” “Sg,” “Rbc,” “Rbcc,” “Htn,” “Wbcc,” “Pot,” and “Bp.” Each classifier was trained on the entire training set using selected top 10 features, and it was subsequently validated on test set. Stratified 5-fold and grid search CV were utilized to extract best values for parameters. Then utilizing these best parameters for each classifier, voting classifier's performance was evaluated through soft voting technique. The best hyperparameters obtained for the approaches using grid-based search CV are stated below.

***K*-NN:** {'n_neighbors': 30, 'weights': 'distance'}, **Logistic Regression:** {'C': 10, 'penalty': 'l2'}, **Random forest:** {'max_depth': 30, 'max_features': 'sqrt', 'min_samples_leaf': 4, 'min_samples_split': 10, 'n_estimators': 200}, **SVM:** {'C': 10, 'gamma': 'scale', 'kernel': 'linear'}, **Decision Tree:** {'criterion': 'gini', 'max_depth': 5, 'min_samples_leaf': 1, 'min_samples_split': 2}, **Gradient Boosting:** {'learning_rate': 0.1, 'max_depth': 3, 'n_estimators': 200}.

The evaluation measures obtained for eight ML models to assess the predictability performance are presented in Table 2 . From Table 2 , it can be deducted that in identifying “CKD” cases, GB obtained the highest accuracy with 98%, followed by voting classifier and decision tree with 97% accuracy. RF performed well achieving 96% accuracy, 98% recall, and low bias value of 0.009. Both SVM and LR obtained 94% accuracy, 94% precision, 98% recall, and 96% F1 score, showing a very good trade-off between false positives and false negatives. *K* -NN and NB achieved 93 and 91% accuracy, respectively. But all the models showed very good accuracy of 91% to over 98%. All classifiers achieved precision, recall, AUC, and F1 score of over 90%.

**Table 2:** Performance analysis of ML models using top 10 features

ML models	Testing accuracy (%)	Precision (%)	Recall (%)	F1 score (%)	AUC	Training loss	Test loss	Bias
***K*** **-NN**	93	92	98	95	0.98	0.002	0.202	0.038
**Logistic regression**	94	94	98	96	0.98	0.193	0.215	0.025
**Random forest**	96	97	98	97	0.99	0.092	0.134	0.009
**Support vector machine**	94	94	98	96	0.98	0.205	0.244	0.029
**Decision tree**	97	98	97	98	0.99	0.103	0.123	0.006
**Gradient boosting**	98	98	99	98	0.99	0.008	0.09	0.003
**Naïve Bayes**	91	97	90	93	0.97	0.551	0.374	0.048
**Voting (soft voting)**	97	98	98	98	0.99	0.088	0.135	0.003

**Fig. 5:**
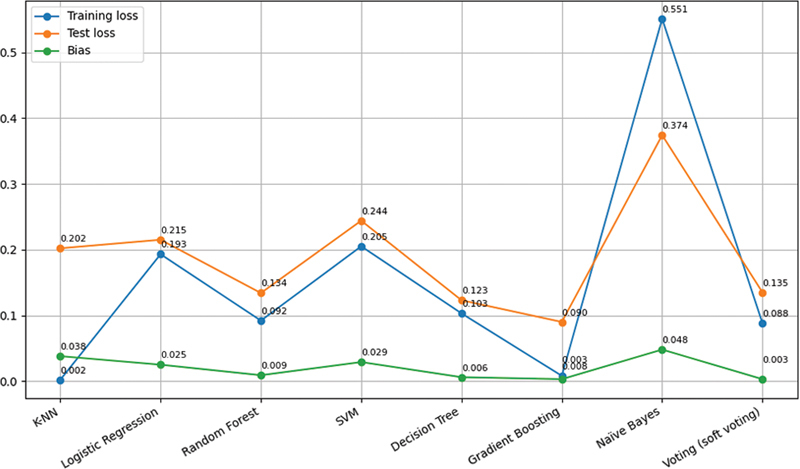
Visualization of training loss, test loss, and bias for eight ML classifiers.

**Fig. 6:**
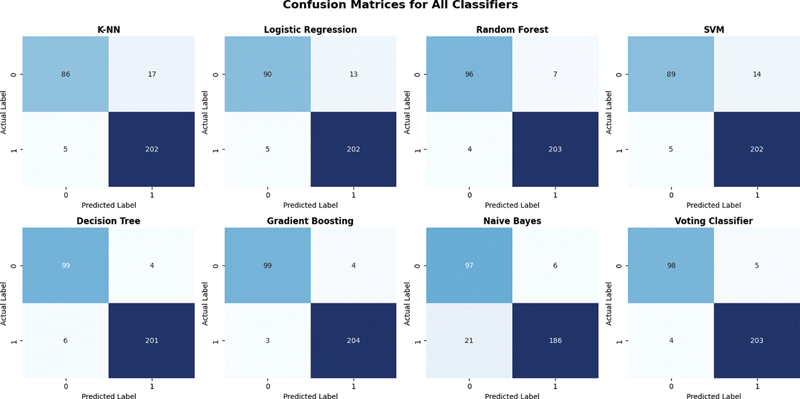
Confusion matrices for all classifiers using feature importance with sequential feature selection (SFS). Here the number in the figures represents number of subjects.

Figure 5 shows the visualization of training loss, test loss, and bias for all classifiers. All the classifiers showed a very minimal amount of bias. Further, the specificity for ML algorithms was calculated separately. The term specificity is a measure of how well a test can identify true negatives. Specificity obtained for ML algorithms: *K* -NN, 83%; LR, 87%; random forest, 93%; support vector machine, 86%; decision tree, 96%; gradient boosting, 96%; Naïve Bayes, 94%; and voting, 95%. Gradient boosting and decision tree showed the highest excellent specificity of 96%, indicating that these could minimize false alarms and give reliable negative predictions. *K* -NN showed the lowest specificity among all the classifiers indicating that 83% were correctly identified as negative, and 17% were incorrectly labeled as positive. Figure 6 shows the confusion matrices for all classifiers indicating how well the algorithms could classify the CKD and non-CKD classes.

### Performance Analysis Using ReliefF Feature Selection

**Fig. 7:**
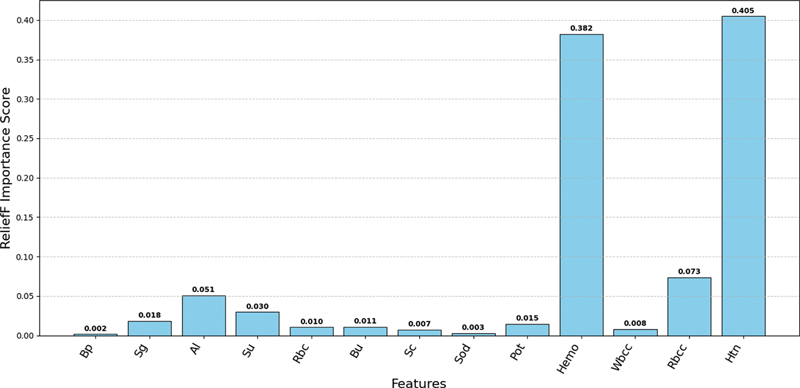
Feature importance score using ReliefF feature selection algorithm.

The ReliefF importance score obtained for 13 features is shown in Fig. 7 . Based on the score, “Htn” (hypertension) is determined to be the significant predictor among these features with importance score 0.405. “Hb” (hemoglobin) was marked as second most important risk factor achieving an importance score of 0.382 followed by “Rbcc” (red blood cells count), “Al” (albumin), “Sg” (specific gravity), “Pot” (potassium), “Bu” (blood urea), “Rbc” (red blood cells), and “Wbcc” (white blood cells count). After this, stratified 5-fold and grid search CV were employed to identify the best parameters. The best hyperparameters acquired for these models using grid-based search CV are presented here.

***K*-NN:** {'n_neighbors': 30, 'p':1, 'weights': 'distance'}, **Logistic Regression:** {'C': 10, 'penalty': 'l2'}, **Random Forest:** {'max_depth': 10, 'min_samples_split': 2, 'n_estimators': 200}, **SVM:** {'C': 10, 'gamma': 'scale', 'kernel': 'linear'}, **Decision Tree:** {'max_depth': 20, 'min_samples_leaf': 5, 'min_samples_split': 5}, **Gradient Boosting:** {'learning_rate': 0.1, 'max_depth': 5, 'n_estimators': 100}

Table 3 shows that all the models had accuracy over 85%. GB achieved the highest accuracy of 98%, followed by ensemble soft voting and RF with 97% accuracy, and DT with 95% accuracy. LR showed a very good performance having 94% accuracy, 98% recall, 96% F1 score, and 94% precision. SVM showed 94% accuracy with a very high recall of 98%. NB showed the lowest performance in terms of accuracy, recall, and F1 score. The bias of the classifiers varied between “0.006 and 0.103.”

**Table 3:** Performance analysis of ML classifiers using ReliefF algorithm

ML models	Testing accuracy (%)	Precision (%)	Recall (%)	F1 score (%)	Training loss	Test loss	Bias
***K*** **-NN**	87	85	98	91	0.001	0.251	0.103
**Logistic regression**	94	94	98	96	0.193	0.215	0.029
**Random forest**	97	98	98	98	0.038	0.118	0.006
**Support vector machine**	94	93	98	95	0.203	0.244	0.032
**Decision tree**	95	95	99	97	0.063	0.064	0.025
**Gradient boosting**	98	98	99	99	0.047	0.081	0.006
**Naïve Bayes**	86	96	83	89	0.427	0.435	0.093
**Voting (soft voting)**	97	97	98	98	0.077	0.137	0.006

The specificity of the classifiers was assessed additionally. Specificity obtained for ML algorithms: *K* -NN, 65%; LR, 86%; random forest, 95%; support vector machine, 85%; decision tree, 89%; gradient boosting, 96%; Naïve Bayes, 93%; and voting, 95%. *K* -NN algorithm significantly underperformed all other models indicating a high rate of false positives since it incorrectly labeled the truly healthy patients as having CKD 35% of the time. The classifiers with high specificity (lower rate of false positives) like gradient boosting, random forest, Naïve Bayes, and voting can be effectively used for ruling out the risk of CKD.

**Fig. 8:**
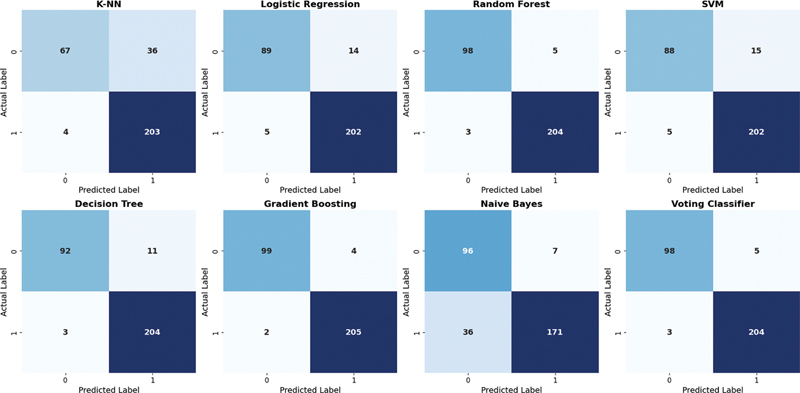
Confusion matrices of all classifiers using ReliefF algorithm. Here the number in the figures represents number of subjects.

Figure 8 shows the confusion matrix of all eight ML classifiers. From the figure it is evident that, classifiers GB, RF, and voting showed a very good performance with only few misclassifications. Out of 310 patient records, SVM classified 88 non-CKD samples correctly but 15 were misclassified as CKD case. Also, 202 CKD cases were correctly labeled as CKD while 5 samples were incorrectly predicted as class non-CKD, although they were actually CKD cases.

**Fig. 9:**
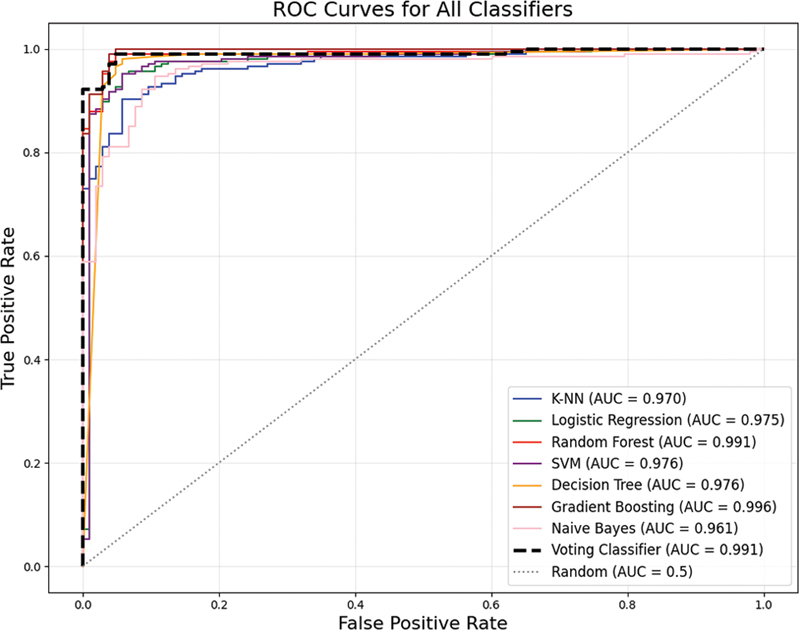
AUC-ROC curve analysis of ML classifiers.

Figure 9 shows that all the ML models efficiently differentiated between negative and positive cases achieving high AUC of over 0.96. GB achieved the highest AUC of 0.996 making it the best model since it also achieved best accuracy, precision, recall, F1 score, specificity, and lowest bias.

### Performance Analysis of ML Classifiers Using Raw Features Without Utilizing Grid-based Search CV

In Table 4 , the performance of ML models is assessed using all features and without utilizing grid search CV. From the table, it is evident that when all features were used without grid search CV, the performance of some algorithms dropped in terms of accuracy, precision, recall, F1 score, and specificity. *K* -NN achieved 85% accuracy when all features were used whereas it achieved 93 and 87% accuracy using top 10 features selected through feature importance with SFS and ReliefF algorithm, respectively, following grid search CV and hyperparameter tuning. The accuracy, precision, recall, and F1 score hadn't changed much for random forest and gradient boosting using all features without grid search CV because of their ensemble architecture and regularization mechanisms which naturally make them robust. But the specificity of *K* -NN, LR, and NB dropped significantly when all raw features were used.

**Table 4:** Performance analysis of ML models using raw features and without grid search CV

ML models	Testing accuracy (%)	Precision (%)	Recall (%)	F1 score (%)	Specificity (%)
*K-* NN	85	85	94	89	67
Logistic regression	93	92	97	95	83
Random forest	97	98	98	98	95
Support vector machine	91	90	97	93	79
Decision tree	94	94	97	96	88
Gradient boosting	98	98	99	98	96
Naïve Bayes	89	95	88	91	90
Voting (soft voting)	97	97	99	98	93

### Comparative Analysis of ML Algorithms' Performance

**Fig. 10:**
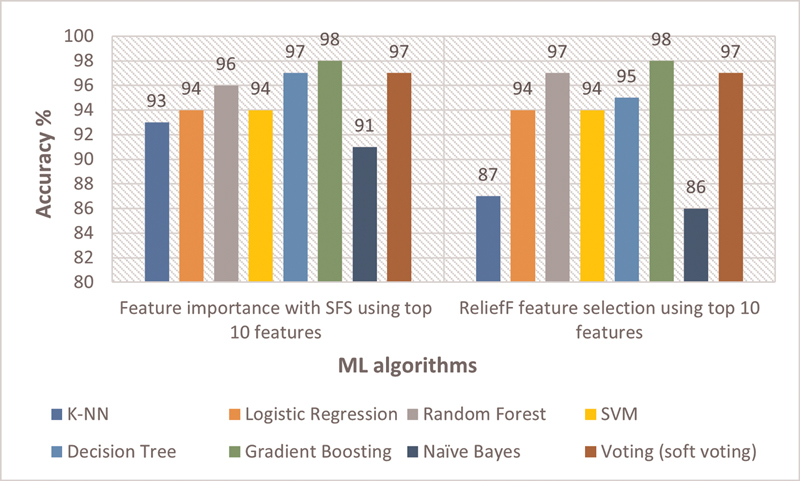
Performance comparison of ML algorithms using top 10 features.

Summary of comparative performance of ML models in terms of accuracy using feature importance with sequential feature selector and ReliefF algorithm is presented in Fig. 10 . On the y-axis, accuracy % is shown while on x-axis the name of the ML models is presented. From this analysis it is easier to infer that all eight models showed a very good performance to predict CKD detection achieving higher accuracies of over 86%. GB showed the highest accuracy of 98% in terms of both feature selection techniques followed by voting classifier (soft voting) achieving an accuracy of 97%.

Table 5 shows the comparative analysis of prior studies for CKD prediction using feature selection techniques. Majid et al used UCI CKD dataset where Naïve Bayes algorithm with chosen features achieved precision of 94.2%, whereas SVM with chosen features showed precision and recall of 97.3 and 93.8%, respectively. 10 In our proposed methodology when feature importance with SFS was used, NB showed a precision of 97% and SVM showed a very good recall at 98%.

**Table 5:** Comparison with previous CKD ML studies

Study	Dataset	ML algorithms with feature selection	Accuracy (%)	Precision (%)	Recall (%)	F1 score (%)
Majid et al 10	UCI CKD dataset	Naïve Bayes and Ranker with attributes chosen	93.4	94.2	93.8	93.8
		SVM and Ranker with InfoGainAttributeEval	97.1	97.3	93.8	97.3
		K-NN and Ranker with attributes chosen	97	97	97	97
Hema et al 25	Real-time dataset	K-NN with exhaustive feature selection	86	74	86	80
		Random forest with exhaustive feature selection	86	74	86	80
		Decision tree with exhaustive feature selection	78	72	74	73
		Gradient boosting with exhaustive feature selection	80	74	84	79
Islam et al 11	CKD- focused dataset	Naïve Bayes	93.9	94	93.9	93.9
		Simple logistic	94.76	94.8	94.8	94.8
		Random forest	98.8	98.9	98.9	98.9
**Proposed methodology**	**CKD- focused dataset**	***K*** **-NN with feature importance with SFS**	**93**	**92**	**98**	**95**
		**Random forest with feature importance with SFS**	**96**	**97**	**98**	**97**
		**SVM with feature importance with SFS**	**94**	**94**	**98**	**96**
		**Decision tree with feature importance with SFS**	**97**	**98**	**97**	**98**
		**Gradient boosting with feature importance with SFS**	**98**	**98**	**99**	**98**
		**Naïve bayes with feature importance with SFS**	**91**	**97**	**90**	**93**

Hema et al used a real-time dataset and exhaustive feature selection technique for predicting CKD. 25 In their proposed methodology, *K* -NN and random forest achieved highest accuracy and recall at 86%. *K* -NN, RF, DT, and NB in our proposed model with both feature selection techniques outperformed in terms of accuracy, precision, recall, and F1 score compared with those models in this paper.

Islam et al utilized decision stump, linear regression, and simple linear regression model for ranking and selecting important features on the CKD-focused dataset. 11 In this paper, NB achieved precision and F1 score of 94 and 93.9%, respectively. With our proposed methodology using feature importance with SFS, NB achieved 97 and 93% of precision and F1 score, respectively.

## Discussion

To summarize, previously there had been a few works on CKD prediction using UCI CKD dataset. This dataset was originally created in 2015, being older than our CKD-focused dataset. The number of the patient records in our dataset is more than double compared with UCI CKD dataset. Also, this small dataset contains several missing values whereas our dataset has only one column with four missing values.

In our proposed methodology, there are some selected data-driven risk factors which are common such as “Htn” (hypertension), “Hemo” (hemoglobin), “Rbcc” (red blood cells count), “Pot” (potassium), “Bu” (blood urea), and “Wbcc” (white blood cells count) using both feature selection techniques. Based on the ranking for both feature selection procedures, hemoglobin is determined to be the significant predictor among these features. Lower than normal level of hemoglobin and red blood cells count can lead to the medical condition called anemia. Anemia is a common complication in CKD, which develops primarily due to reduced renal synthesis of erythropoietin. 6 Although it is less visible in early stages of CKD but it often gets worse as the disease progresses and kidney function is lost substantially. Therefore, these two risk factors, “Hemo” (hemoglobin) and “Rbcc” (red blood cells count), can play an important role in the diagnosis and prediction of CKD. Our one of the most important selected features is hypertension, whose high prevalence is also considered as a well-known factor for influencing the progression of CKD. 2

Another important identified risk factor is blood urea. The kidney's function is to filter urea (a chemical waste product) from blood, and high levels of urea in the blood can indicate decrease in renal clearance resulting in acute and chronic renal failure or impairment. 4 Along with the risk factor blood urea, lower than normal albumin content in blood also plays a vital role in CKD screening. 4 Prior identification of all these mentioned important risk factors may subsequently help medical experts to make precise early identification of CKD.

In prior studies, authors used several algorithms with different feature selection techniques on a variety of datasets. They assessed the performance of the model using accuracy, precision, recall, and F1 score. In our proposed methodology, along with these evaluation measures we also focused on finding specificity, training loss, test loss, and bias, which can ensure accurate and reliable diagnosis of CKD in clinical setting. Also, in our study we explicitly identified the risk factors based on ranking score instead of only using them for improving a model's performance metrics.

It is evident that gradient boosting outperformed other seven models, achieving the highest accuracy, precision, recall, F1 score, and specificity. It also achieved low bias because of its property of building trees sequentially where each new tree corrects the errors of the previous ones. Random forest also performed very well in terms of several evaluation measures because ensemble classifiers are efficient in handling heterogenous data, nonlinearity, and complex feature interactions. Even decision tree gave good results although it is not an ensemble classifier. Some models like *K* -NN and NB underperformed because these basic models are very simple in nature. *K* -NN searching method relies on distance metrics and is not efficient like tree-based models in terms of handling heterogenous and multivariate data, which typically can lead to unreliable outcome. Naïve Bayes underperforms because of its unrealistic independent assumptions about features which is very crucial in clinical data.

Nevertheless, some of the ML approaches like GB, RF, DT, and voting classifier showed a very good result in terms of accuracy, recall, precision, and F1 score with values over 95%. These classifiers also achieved high specificity of over 93%, demonstrating their ability to identify the negative cases quite correctly.

## Conclusion

Since the size of the dataset is not that large, we chose DT, RF, and GB as they are very well suited to capture patterns for the dataset, reduce overfitting by combining multiple DTs, and learn sequentially by handling the datasets efficiently. The chosen methods follow different principles: *K* -NN searches with various distance measures, and Naïve Bayes assumes independence between features. Soft voting was used here since it often produces better results than hard voting because it considers the confidence levels of the classifiers on the same dataset and first trains several ML models with selected features. Neural networks and other more complex classification algorithms were not chosen as these could require more data and more extensive computational resources to build an effective model. So, it can be concluded that in the suggested methodology, the two notable feature selection techniques identified the important risk factors, and the classifiers showed a very good performance in estimating CKD's presence or absence with greater accuracy and other evaluation measures.

Therefore, it can be inferred that utilizing the proposed methodology, individuals at risk of developing chronic renal disease can be detected earlier more accurately than with traditional methods. This certainly holds a great promise toward enhancing healthcare judgment and eventually ensuring treatment for patients.

## Methodological Limitation and Future Scope

Currently, our proposed methodology utilizes only multivariate tabular clinical data for comprehensive analysis. It does not address how feature selection techniques like ReliefF or feature extraction techniques can be effectively used in real-time and image dataset for accurate prediction of CKD earlier. Among the algorithms that we used here, tree-based models performed really well compared with basic algorithms like *K-* NN and NB. So, in future we must focus separately on the implementation of these simple models for the improvement of their robustness so that these may handle heterogenous, noisy, and scaled data, and provide more accurate yet realistic outcome.

In future, we aim to extend our work for early and accurate diagnosis of CKD from multimodal datasets. Furthermore, collaborating with experts in medical laboratories for analyzing clinical data, biomarkers, CT, X-rays, and MRI scans using ML and DL approaches can play a vital role in identifying CKD cases along with precisely identifying the stage of the disease. This will lead to more individualized patient care. Additionally, for future research, exploration of risk factors associated with different types of CKD including IgA nephropathy and lupus nephritis, and early prediction of those indicators to enhance predictive performance can be considered.

## References

[1] Foreman K J Marquez N Dolgert A Forecasting life expectancy, years of life lost, and all-cause and cause-specific mortality for 250 causes of death: reference and alternative scenarios for 2016-40 for 195 countries and territories Lancet 2018 392(10159):2052 2090 30340847 10.1016/S0140-6736(18)31694-5 PMC6227505

[2] American Society of Nephrology European Renal Association International Society of Nephrology Francis A Harhay M N Ong A CM Chronic kidney disease and the global public health agenda: an international consensus Nat Rev Nephrol 2024 20 07 473 485 38570631 10.1038/s41581-024-00820-6

[3] Chronic Kidney Disease in the United States 2023 Centers for Disease Control and Prevention. Accessed March 29, 2025 at:https://www.cdc.gov/kidneydisease/publications-resources/CKD-national-facts.html

[4] Gounden V Bhatt H Jialal I Renal function tests Treasure Island, FL StatPearls Publishing 2024 29939598

[5] Jager K J Kovesdy C Langham R Rosenberg M Jha V Zoccali C A single number for advocacy and communication-worldwide more than 850 million individuals have kidney diseases Nephrol Dial Transplant 2019 34 11 1803 1805 31566230 10.1093/ndt/gfz174

[6] Arora P Chronic Kidney Disease (CKD) Clinical Presentation. MedscapeAccessed March 31, 2025 at:https://emedicine.medscape.com/article/238798-clinical

[7] Arif M S Mukheimer A Asif D Enhancing the early detection of chronic kidney disease: a robust machine learning model Big Data Cogn Comput. 2023 7 03 144

[8] Qezelbash-Chamak J Badamchizadeh S Eshghi K Asadi Y A survey of machine learning in kidney disease diagnosis Mach Learn Appl 2022 10 100418

[9] Wang W Chakraborty G Chakraborty B Predicting the risk of chronic kidney disease using machine learning algorithm Appl Sci (Basel) 2020 11 01 202

[10] Majid M Gulzar Y Ayoub S Using ensemble learning and advanced data mining techniques to improve the diagnosis of chronic kidney disease Int J Adv Comput Sci Appl 2023 14 10 470 480

[11] Islam M A Akter S Hossen M S Keya S A Tisha S A Hossain S Risk factor prediction of chronic kidney disease based on machine learning algorithmsIn: Proceedings of the 2020 3rd International Conference on Intelligent Sustainable Systems (ICISS); Dec 3–5 2020; Thoothukudi, India. IEEE;2020 952 957

[12] Bravin P S A review on data preprocessing techniques in data mining Int J Adv Eng Manag 2022 4 05 1206 1209

[13] Adil I H Zaman A Outliers detection in skewed distributions: Split sample skewness based boxplot Econ Comput Econ Cybern Stud Res 2020 03 279 296

[14] Sequential Feature Selection. GeeksforGeeksAccessed March 31, 2025 at:https://www.geeksforgeeks.org/sequential-feature-selection/

[15] Robnik-Šikonja M Kononenko I Theoretical and empirical analysis of ReliefF and RReliefF Mach Learn 2003 53 23 69

[16] Suthaharan S Support vector machine Cham Springer 2016 207 235

[17] Sumathi S Rajappa S Kumar L A Paneerselvam S Machine Learning for Decision Sciences with Case Studies in Python Boca Raton, FL CRC Press 2022

[18] Damanik I S Windarto A P Wanto A Andani S R Saputra W Decision tree optimization in C4.5 algorithm using genetic algorithm J Phys Conf Ser 2019 1255 01 12012

[19] Pandey R Maurya P Chiong R Data Modelling and Analytics for the Internet of Medical Things Boca Raton, FL CRC Press 2023

[20] Reddy E MK Gurrala A Hasitha V B Kumar K VR Introduction to Naive Bayes and a review on its subtypes with applications Singapore Springer 2022 1 14

[21] Nusinovici S Tham Y C Chak Yan M Y Logistic regression was as good as machine learning for predicting major chronic diseases J Clin Epidemiol 2020 122 56 69 32169597 10.1016/j.jclinepi.2020.03.002

[22] Belyadi H Haghighat A Machine Learning Guide for Oil and Gas Using Python: A Step-by-Step Breakdown with Data, Algorithms, Codes, and Applications Houston, TX Gulf Professional Publishing 2021

[23] Kabari L G Onwuka U C Comparison of bagging and voting ensemble machine learning algorithm as a classifier Int J Adv Res Comput Sci Softw Eng 2019 9 03 19 23

[24] Ranjan G SK Verma A K Radhika S K-nearest neighbors and grid search CV based real-time fault monitoring system for industriesIn: Proceedings of the 2019 IEEE 5th International Conference for Convergence in Technology (I2CT);2019; Bombay, India. pp 1–5

[25] Hema K Meena K Pandian R Analyze the impact of feature selection techniques in the early prediction of CKD Int J Cogn Comput Eng 2024 5 66 77

